# Transcriptome Analysis of Wild *Bletilla striata* Tubers Across Multiple Years Revealed the Molecular Mechanisms Regulating Polysaccharide Metabolism and Tuber Enlargement

**DOI:** 10.3390/plants14050689

**Published:** 2025-02-24

**Authors:** Hai Huang, Long Yang, Chunfang Luo, Tuo Qi, Junna Duan

**Affiliations:** 1 Guizhou Institute of Subtropical Crops, Guizhou Academy of Agricultural Sciences, Xinzhong Road, Huaxi District, Guiyang 550000, China; yellowsea87@126.com (H.H.); yanglong0913@163.com (L.Y.); 15870376858@163.com (C.L.); 2 Ecological Security and Protection Key Laboratory of Sichuan Province, Mianyang Normal University, Mianyang 621000, China

**Keywords:** *Bletilla striata*, polysaccharide, functional traits, regulate genes

## Abstract

A *Bletilla striata* (Thunb.) *Reichb*.*f*., known as Bai Ji in Chinese, is a plant from the Orchidaceae family that has been used for its medicinal properties for thousands of years in China. *B. striata* holds significant economic value due to its esteemed medicinal applications. Our study aimed to analyze the transcriptome of wild *B. striata* tubers across multiple years to understand the molecular mechanisms regulating polysaccharide metabolism and tuber enlargement. We collected wild *B. striata* samples of different growth ages and analyzed their chemical composition, including total phenols, polysaccharides, alkaloids, and proteins. The results showed that the content of these compounds varied with the growth age of the plants. Our study focused on analyzing the genes associated with growth years and accelerating the seedling growth cycle, which holds immense value for the preservation and optimal utilization of superior *B. striata* orchid resources. To further investigate the underlying molecular mechanisms, we performed a comprehensive transcriptome analysis to explore gene expression, functional annotation, and regulatory networks related to the development and chemical composition of *B. striata* tubers. The quality of perennial medicinal herbs is intricately linked to their growth age. Unfortunately, excessive wild resource excavation has resulted in the premature harvesting of these herbs, causing a decline in their overall quality and effectiveness. Our study offers valuable insights into the conservation and utilization of *B. striata* resources.

## 1. Introduction

*Bletilla striata*, a perennial herbaceous plant from the Orchidaceae family, is a valuable Chinese medicinal herb with a history spanning over two millennia [1,2]. Thriving in warm, humid environments such as hilly areas and valleys near vegetation and streams, it thrives in soil rich in organic matter for optimal growth. Among the four Chinese *Bletilla* species—*Bletilla sinensis*, *Bletilla formosana*, *Bletilla striata*, and *Bletilla ochracea*—key production regions include Guizhou, Sichuan, and Hunan [1,3,4]. Featuring ovoid or irregular pseudobulbs as nutrient and water storage organs, *Bletilla striata* is esteemed for its medicinal properties [5,6]. These pseudobulbs are rich in polysaccharides [7], dihydrophenanthrenes [8,9], steroids, triterpenes, volatile oils [10], and other bioactive compounds, notably glucomannan, a key mucilaginous component forming a gel with water [11]. This gel is renowned for its efficacy in reducing swelling and arresting bleeding, commonly applied in treating hemoptysis, hematemesis, wounds, abscesses, and skin conditions [1,3,5,12]. Additionally, *Bletilla* demonstrates diverse pharmacological activities such as antibacterial, anti-inflammatory, antioxidant, immune-modulating, anti-fibrotic, and anti-aging effects [13,14,15,16,17,18].

*B.striata* not only holds high medicinal value but also possesses significant ornamental and economic value [2,19,20]. Due to their beautiful flower morphology and diverse colors, Bletilla flowers are highly ornamental and extensively used in landscaping for gardens. *Bletilla* is a crucial raw material in modern pharmaceutical and cosmetic industries due to its long-lasting skincare effects, stable performance, mild action, low irritation, and high safety factor [21]. *B. striata* is also widely used as an adhesive in high-end cigarette production and as a repairing agent for broken roots of Panax ginseng [22]. Due to its significant medicinal, ornamental, and economic values, *B. striata* has traditionally relied on wild resources, leading to overexploitation and indiscriminate digging, resulting in the endangered status of wild resources [4].

*B. striata* contains rich chemical components, including phenolic acids, biphenyls, flavonoids, triterpenoids, steroids, polysaccharides, and terpenes, and these compounds endow *B. striata* with a wide range of pharmacological activities [8,9,13,15,16,18,22]. Polysaccharides, prominently found in *B. striata*, offer a spectrum of medicinal benefits [2]. *B. striata* polysaccharides (BSPs) serve as immune modulators, aiding in gastric ulcer prevention and coagulation promotion [23]. The functionality and structure of BSPs are crucial for their bioactivities, rendering them valuable biomaterials for drug delivery and wound healing applications through crosslinking or modification with other substances [10,13,15,22]. Despite the diverse biological roles of BSPs, the intricate biosynthetic pathway of polysaccharide production remains partially understood due to limited genetic information on BSPs. Therefore, comprehensive sequencing studies are imperative to elucidate the genetic composition of BSPs, unveiling insights into BSP genes and their functions.

## 2. Results

### 2.1. Effects of Growth Ages on B. striata Growth and Development

**Figure 1:**
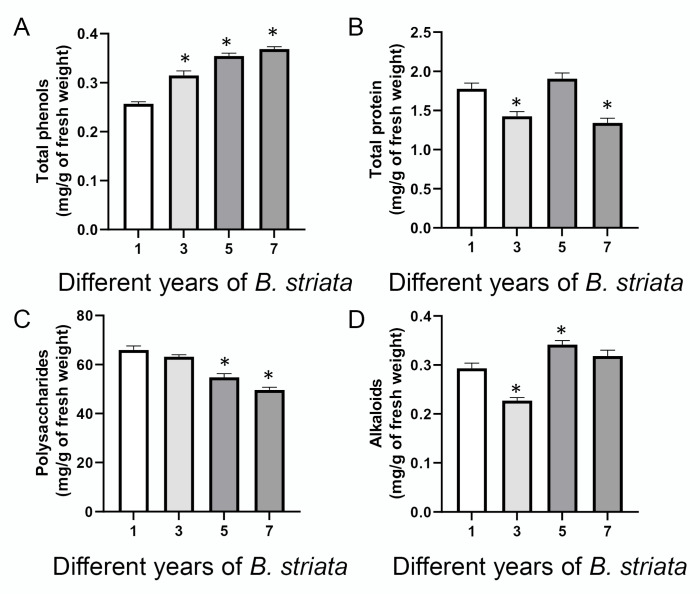
The growth and development of *B. striata* under different growth years. (**A**) Total phenols contents (mg/g of fresh weight) of 1, 3, 5, and 7 years in rhizome of *B. striata* orchid. (**B**) Total protein contents (mg/g of fresh weight) of 1, 3, 5, and 7 years in rhizome of *B. striata* orchid. (**C**) Polysaccharide contents (mg/g of fresh weight) of 1, 3, 5, and 7 years in rhizome of *B. striata* orchid. (**D**) Alkaloids contents (mg/g of fresh weight) of 1, 3, 5, and 7 years in rhizome of *B. striata* orchid. Deviation of values obtained from three independent biological replicates. Values were tested according to statistical *t*-test. * Indicates a significant difference, with a *p*-value less than 0.05.

*Bletilla striata* is an important Chinese herbal plant widely cultivated in southwest China. The growth age of perennial medicinal plants is crucial for the quality of medicinal herbs. We collected *B. striata* samples of various growth ages from Guizhou province (Figure S1) and analyzed the levels of total phenols, polysaccharides, alkaloids, and total proteins (Figure 1). The total phenols content of *B. striata* increases steadily with age. The phenol content in *B. striata* bulbs shows a progressive increase over time: beginning at 0.257 mg/g in the first year, increasing to 0.31 mg/g in the third year, peaking at 0.35 mg/g in the fifth year, and slightly rising to 0.368 mg/g by the seventh year. The protein content in *B. striata* bulbs fluctuates over the years: beginning at 1.77 mg/g in the first year, decreasing to 1.43 mg/g in the third year, rising to 1.9 mg/g in the fifth year, and subsequently dropping to 1.34 mg/g by the seventh year. The polysaccharide content of *B. striata* exhibits a decline over the years: starting at 65.93 mg/g in the first year, decreasing to 63.13 mg/g in the third year, dropping further to 54.8 mg/g in the fifth year, and reaching 49.63 mg/g by the seventh year. The total alkaloid content of *B. striata* shows variability over the years: starting at 0.29 mg/g in the first year, decreasing to 0.23 mg/g in the third year, increasing to 0.34 mg/g in the fifth year, and stabilizing at 0.32 mg/g by the seventh year. *B. striata* samples from various years exhibit a discernible pattern in physiological indicators; however, accurately determining the growth years and medicinal properties of the herb remains challenging. Hence, additional research is essential to identify molecular evaluation markers for enhanced precision.

### 2.2. RNA Sequencing and Assembly

In this study, RNAseq analysis was conducted on 12 samples using the Illumina NovaSeq platform. The results showed the generation of 672,466,332 bp of raw reads and 660,320,380 bp of clean reads (see Supplementary Table S2). Each sample demonstrated Q20 and Q30 values exceeding 99% and 97%, respectively, with an error rate below 0.01% and GC content ranging from 46.54% to 47.44%. Transcripts were assembled, de-redundant, and utilized as reference sequences for aligning clean reads from each sample, resulting in mapped reads ranging from 85.81% to 88.76% and unique reads ranging from 20.35% to 21.11%. Utilizing the high-quality sequencing data, functional annotations were assigned to 253,550 transcripts and 127,294 unigenes (see Supplementary Table S2). Subsequently, the unigenes were annotated against various databases, including the Non-Redundant Protein (NR) Database, Gene Ontology (GO), Kyoto Encyclopedia of Genes and Genomes (KEGG), Evolutionary Genealogy of Genes: Non-supervised Orthologous Groups (eggNOG), Swiss Institute of Bioinformatics (Swiss-Prot), and Database of Protein Families (Pfam). Of these, 81,516 unigenes (64.04% of total unigenes) were annotated to the NR database, while 67,420 (52.96%), 60,862 (47.81%), 53,592 (42.1%), 48,419 (38.04%), 80,173 (62.98%), and 54,620 (42.91%) unigenes were annotated to the GO, KEGG, Pfam, clusters of orthologous groups for eukaryotic complete genomes (KOG), translation from EMBL (TrEMBL), and Swiss-Prot databases, respectively (Supplementary Table S2).

To visually compare gene expression levels across samples, box plots were employed to illustrate the distribution of gene expression within individual samples (Figure S2A). The consistency in expression levels among samples suggests that there are no sequencing quality issues. To identify truly significant differentially expressed genes, it is crucial to address expression variations due to biological diversity and assess the significance of biological replicates. The correlation statistics among samples in this study are presented in Figure S2B. Samples from the same year exhibit a similarity exceeding 92%. Additionally, samples from different years show a similarity of approximately 80%. Unsupervised pattern recognition utilizing principal component analysis (PCA) was employed to effectively differentiate ginseng samples. This method condenses multi-index data into a few feature components, offering visual representations of significant sample distinctions (Figure S2C). By clustering genes with similar expression patterns, the identification of unknown gene functions or the unexplored functions of known genes becomes feasible. Genes in the same cluster often exhibit similar functions or partake in common metabolic processes or cellular pathways. Hierarchical clustering analysis was performed on differentially expressed genes within each comparison group. The data were standardized using Z-scores, leading to the creation of a clustered heatmap displaying all differential genes across comparison groups and individual heatmaps for each specific comparison group (depicted in Figure S2D). Significant differences in gene expression between different years can be used as a reference for detecting the growth years of *B. striata*.

### 2.3. KEGG Pathway Analysis of DEGs Under Different Growth Ages

**Figure 2:**
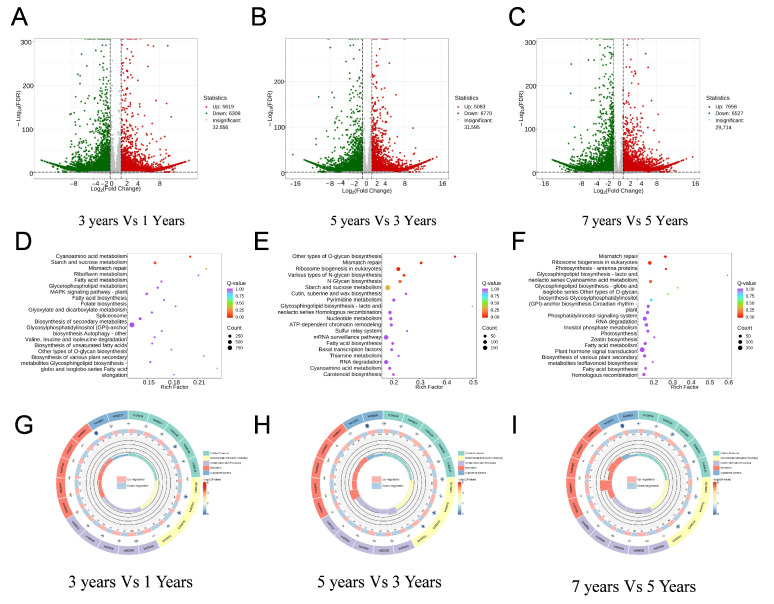
Expression profiling changes and KEGG enrichment bubble diagram of differential genes in *B. striata* for different growth years. (**A**–**C**) Volcano plot of differentially expressed genes; (**D**–**F**) the functions of the DEGs were characterized using Kyoto Encyclopedia of Genes and Genomes (KEGG) pathway; (**G**–**I**) circular plot of differentially expressed gene KEGG enrichment. (**A**,**D**,**G**) 3 years vs. 1 year; (**B**,**E**,**H**) 5 years vs. 3 years; (**C**,**F**,**I**) 7 years vs. 5 years.

In the quest to uncover the genes influencing the growth and development of *B. striata*, differentially expressed genes (DEGs) were identified among the samples using stringent criteria: a *p*-value < 0.05 and |log2 FC (fold change)| ≥ 1 (refer to Supplementary Table S3). In the comparison between the 3-year group and the 1-year group (3 years vs. 1 year), a total of 11,928 DEGs were detected, comprising 5619 upregulated genes (depicted as red dots) and 6309 downregulated genes (depicted as green dots) (Figure 2A). Additionally, in the comparison between the 5-year group and the 3-year group (5 years vs. 3 years), 11,853 DEGs were found, including 5083 upregulated genes (depicted as red dots) and 6770 downregulated genes (depicted as green dots) (Figure 2B). Lastly, a total of 14,185 DEGs were identified, with 7658 upregulated genes (red dots) and 6527 downregulated genes (green dots) in the comparison between the 3-year group and the 1-year group (7 years vs. 5 years) (Figure 2C). A volcano map of the differential gene analysis and heatmaps of the gene clusters with the most significant differences are shown in Figure 2A–C.

To identify the genes influencing the growth and development of *B. striata* bulbs, we analyzed DEGs across various growth stages of *B. striata*. During the progression from 1 year to 3 years of growth age, significantly up-regulated pathways included cyanoamino acid metabolism and Starch and sucrose metabolism (Figure 2D). Subsequently, with the growth age advancing from 3 years to 5 years, significantly up-regulated pathways included other types of O-glycan biosynthesis, mismatch repair, ribosome biogenesis in eukaryotes, various types of N-glycan biosynthesis, N-Glycan biosynthesis, starch and sucrose metabolism, mismatch repair, ribosome biogenesis in eukaryotes, photosynthesis-antenna proteins, glycosphingolipid biosynthesis—lacto and neolacto series, canoamino acid metabolism (Figure 2E). Likewise, as the growth age further increased from 5 years to 7 years, the significantly up-regulated pathways comprised mismatch repair, ribosome biogenesis in eukaryotes, photosynthesis-antenna proteins, glycosphingolipid biosynthesis—lacto and neolacto series, cyanoamino acid metabolism (Figure 2F).

The analysis concentrated on the top five KEGG pathways with the lowest q-values per KEGG_level_1 for generating a KEGG enrichment circle plot. Differentially expressed genes were significantly enriched during the transition from 1 to 3 years in pathways like plant–pathogen interaction (ko04626), starch and sucrose metabolism (ko00500), spliceosome (ko03040), plant hormone signal transduction (ko04075), ABC transporters (ko02010), and endocytosis (ko04144) (Figure 2G). Subsequently, as age progressed from 3 to 5 years, enrichment was notable in pathways such as plant–pathogen interaction (ko04626), endocytosis (ko04144), Plant hormone signal transduction (ko04075), ABC transporters (ko02010), and MAPK signaling pathway—plant (ko04016) (Figure 2H). Advancing from 5 to 7 years, genes were primarily enriched in pathways including plant–pathogen interaction (ko04626), Plant hormone signal transduction (ko04075), ABC transporters (ko02010), endocytosis (ko04144), MAPK signaling pathway—plant (ko04016), and Starch and sucrose metabolism (ko00500) (Figure 2I).

### 2.4. Enrichment Analysis of Differential Genes in Gene Ontology

GO enrichment analysis indicated that as the growth age progressed from 1 year to 3 years, DEGs were enriched in processes such as polysaccharide catabolic process, histone lysine methylation, benzene-containing compound metabolic process, systemic acquired resistance, and response to symbion. As for comparing the 5 growth years with the 3 years *B. striata*, GO enrichment enriched in histone lysine methylation, phosphatidylinositol metabolic process, phosphatidylinositol biosynthetic process, microtubule-based movement, and regulation of mRNA processing terms. As for comparing the 7 growth years with the 5 years *B. striata*, GO enrichment enriched in glycosylation, glycoprotein biosynthetic process, protein glycosylation, macromolecule glycosylation, and epigenetic regulation of gene expression. Additionally, we clustered homologous proteins of *B. striata*. The COG terms were predominantly enriched in posttranslational modification, protein turnover, chaperones, and signal transduction mechanisms (Figure S3A–C).

The analysis focused on the top 5 GO terms with the lowest q-values per term to generate GO term enrichment circle plots. Differentially expressed genes exhibited significant enrichment during the transition from 1 to 3 years in pathways such as protein–DNA complex (GO:0032993), DNA packaging complex details (GO:0044815), polysaccharide catabolic process (GO:0000272), monosaccharide binding (GO:0048029), and structural constituent of chromatin (GO:0030527). When comparing 5 growth years with 3 years of *B. striata*, GO enrichment was observed in organism-specific (GO:0016604), nuclear speck (GO:0016607), organism-specific (GO:0016796), and enzyme activity (GO:0004527). Furthermore, when comparing 7 growth years with 5 years of *B. striata*, GO enrichment was noted in extracellular enzyme activity (GO:0004527), RNA helicase activity (GO:0003724), ATP-dependent activity acting on RNA (GO:0008186), embryo sac development (GO:0009553), and chloroplast inner membrane (GO:0009706) (refer to Figure S3).

**Figure 3:**
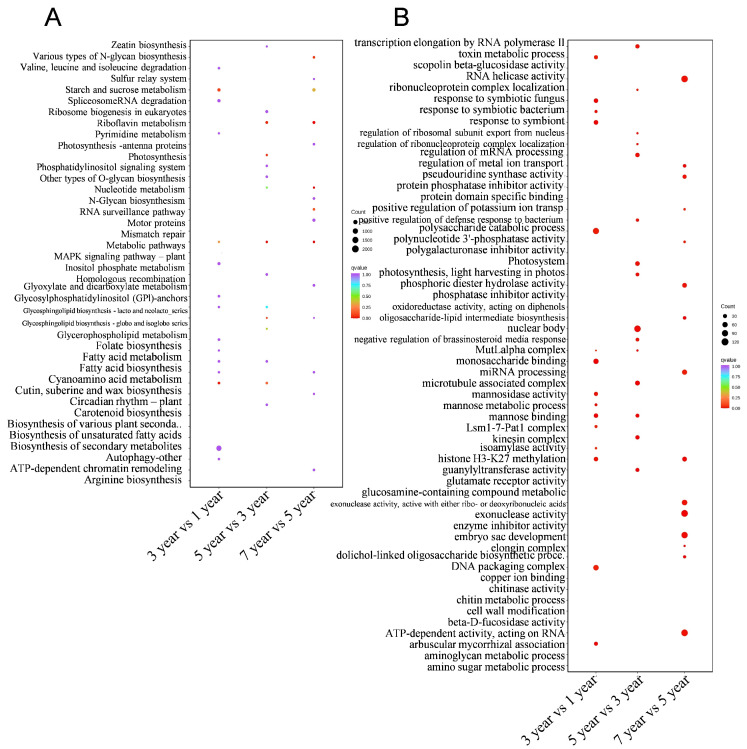
Scatter plot of KEGG enrichment and GO enrichment for multiple groups. The functions of the DEGs were characterized using Gene Ontology (GO) terms (**A**) and Kyoto Encyclopedia of Genes and Genomes (KEGG) pathway (**B**) for multiple groups.

In the analysis involving multiple comparison groups, the KEGG enrichment results of each group were compared, and sorted by q-value, and the top 15 significantly enriched pathways were selected for merging and display, as depicted in Figure 3A. Pathways such as starch and sucrose metabolism, metabolic pathways, cyanoamino acid metabolism, and biosynthesis of secondary metabolites were all enriched as the growth age progressed. Furthermore, upon comparing the GO enrichment results of each comparison group, it was observed that response to symbiotic fungus, response to symbiotic bacterium, response to symbiont, and polysaccharide catabolic process GO terms were all enriched with the advancement of growth age Figure 3B.

Additionally, we annotated and categorized transcription factors (TFs) (Figure S3D–F). Activation of expression was observed in 58 C2H2 TFs (9.15%), 41 AP2/ERF-ERF TFs (6.47%), and 39 NAC TFs (6.15%) as the age progressed from 1 year to 3 years. When comparing 5 growth years with 3 years of *B. striata*, activated expression was seen in 42 bHLH TFs (6.85%), 34 FAR1 TFs (5.55%), 34 AP2/ERF-ERF TFs (5.55%), and 34 C2H2 TFs (5.55%). Furthermore, when comparing 7 growth years with 5 years, activated expression was observed in 39 C2H2 TFs (5.82%) and 36 C3H TFs (5.37%).

### 2.5. Variation in Functional Traits Under Different Growth Ages

In our study of gene expression patterns under various conditions, we first normalized the FPKM values of all differentially expressed genes. Subsequently, K-means clustering analysis was performed to group genes with similar expression patterns across different experimental treatments. Genes within the same cluster are likely to share common functions, as depicted in Figure S4: 1937 DEGs enriched in subclass 9 exhibited specifically high expression in the annual *B. striata*. 3127 DEGs enriched in subclass 5 showed specifically high expression in the 3-year growth stage of *B. striata*. 3542 DEGs enriched in subclass 7 displayed specifically high expression in the 5-year growth stage of *B. striata*. 4924 DEGs enriched in subclass 10 demonstrated specifically high expression in the 7-year growth stage of *B. striata*. DEGs in subclass 4 exhibited a decrease in expression with increasing growth age, while DEGs in subclass 8 showed an increase in expression with growth age.

**Figure 4:**
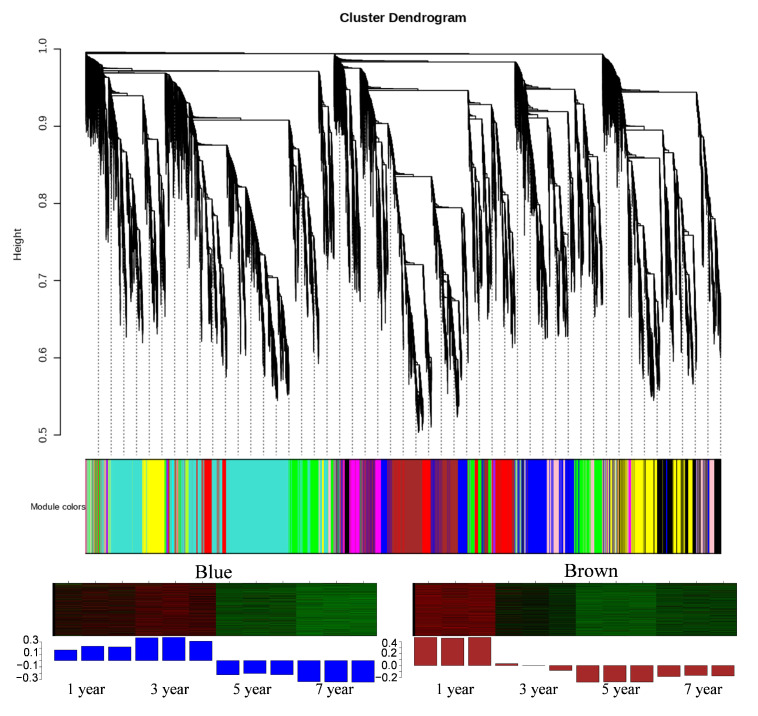
WGCNA analyzes t of *B. striata* under different growth years. (**A**): WGCNA builds a cluster tree according to the correlation between gene expression levels and divides the modules. (**B**): Genes with different expression patterns were clustered in blue, and brown may contribute to the growth and development of *B. striata*.

Additionally, weighted correlation network analysis (WGCNA) will construct a clustering tree to divide modules based on gene expression level correlations. Each color in the figure represents a module where genes cluster together. Genes consistently showing similar expression changes may be functionally related (Figure 4). For instance, 52 DEGs in the blue module were highly expressed in the 1-year and 3-year growth stages of *B. striata* (Table S4), with 15 being hypothetical proteins. Similarly, 52 DEGs in the brown module exhibited high expression in the 1-year growth stage of *B. striata* (Table S5), with 19 being hypothetical proteins. Exploring the functions of genes with unknown roles in *B. striata* can improve our comprehension of its genetic resources.

Microsatellite markers, composed of simple repeat sequences with a core sequence flanked by conserved sequences, are uniformly distributed in eukaryotic genomes. The variability in repeat numbers within the core sequence contributes to their high polymorphism, while the flanking sequences enable specific chromosome localization. SSRs, compared to other markers, offer wide distribution, high polymorphism, codominance, and repeatability. They find extensive use in variety identification, phylogenetic studies, genetic diversity research, linkage mapping, and marker-assisted breeding. Our analysis identified six SSR types: Mono-, Di-, Tri-, Tetra-, Penta-, and Hexa-nucleotide repeats, detailed in Table S6. Despite variable genomic positions, SSR flanking regions typically exhibit conserved single-copy sequences. Primers are designed using these conserved sequences to amplify a single PCR fragment, generating products of varying lengths due to core sequence repeat variations. Gel electrophoresis of PCR products reveals SSR locus polymorphism. SSR primer design was performed using Primer3, with results presented in Table S7. This approach aids in amplifying SSR loci and facilitates their polymorphism display through gel electrophoresis, enhancing applications in genetic research and breeding programs.

### 2.6. Identification of Genes Related to Biosynthesis in Bletilla striata at Different Growth Ages

**Figure 5:**
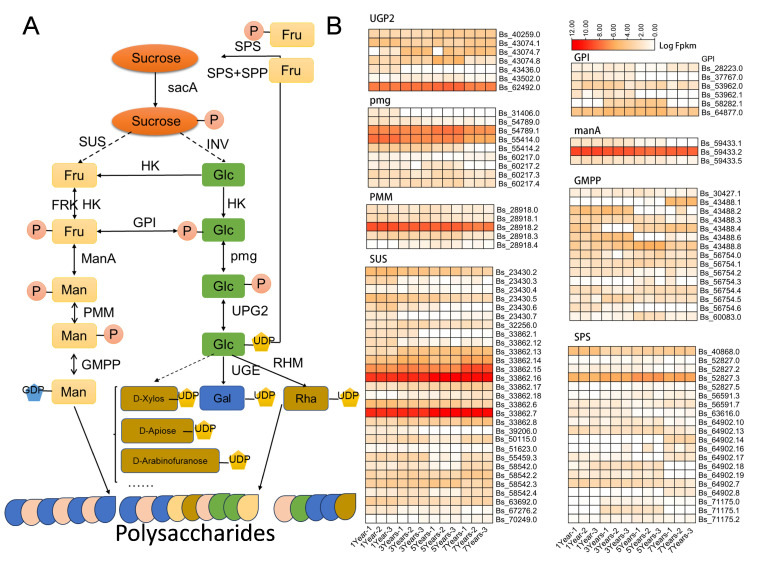
Expression of genes involved in Polysaccharides biosynthesis. (**A**): Diagram showing the Polysaccharides biosynthesis pathways. (**B**): Heatmap analysis illustrating the expression levels of key enzymes (log2-fold change). Fru: Fructose; Man: Mannose; Glc: Glucose; Gal: galactose; Rha: rhamnose; UDP: uridine diphos-phate; HK: hexokinase; INV: invertase; SPS: sucrose phosphate synthase; UPG2: uri-dine-diphosphate glucose pyrophosphorylase; pmg: phosphoglucomutase; GPI: glu-cose-6-phosphate isomerase; ManA: mannose-6-phosphate isomerase; GMPP: GDP-mannose py-rophosphorylase; SUS: sucrose synthase; PMM: phosphomannose isomerase; UGE: UDP-glucose-4-epimerase; FRK: fructokinase; RHM: UDP-rhamnose synthase.

Bletilla striata is abundant in diverse active compounds, such as polysaccharides, flavonoids, saponins, phenols, and lignans, with polysaccharides being the predominant active constituent. The polysaccharides extracted from *B. striata* orchid, known as Bletilla striata polysaccharide (BSP), mainly consist of glucomannan and other mucilaginous substances, which have the property of forming viscous hydrophilic gels when dissolved in water. The hemostatic effect of *B. striata* orchid is closely related to its high content of mucilaginous components. Polysaccharides from *B. striata* orchids find extensive applications in the food and pharmaceutical sectors, showcasing diverse effects like antiviral, antibacterial, antioxidant, and moisturizing properties, highlighting their substantial practical utility. In this study, we analyzed the biosynthesis of polysaccharides. The biosynthetic pathway of *B. striata* polysaccharides primarily encompasses three key processes: the conversion of sucrose, the transformation of uridine diphosphate glucose (UDP-glucose) into other nucleotide diphosphate (NDP) monosaccharides, and the polymerization of polysaccharides. Central to this synthesis are several critical enzymes, including sucrose synthase (SUS), sucrose phosphate synthase (SPS), invertase (INV), hexokinase (HK), fructokinase (FRK), UDP-glucose dehydrogenase (UGDH), UDP-glucose pyrophosphorylase (UGPase), UDP-rhamnose synthase (RHM), phosphomannose isomerase (PMM), GDP-mannose pyrophosphorylase (GMPP), and glycosyl transferases (GTs) (Figure 5A). Based on the transcriptome annotation data, DEGs involved in the BSP pathway were determined in *B. striata* (Figure 5B and Figures S5 and S6), a total of 298 BSP-related enzymes, including seven pyrophosphorylase (UGP2, EC 2.7.7.9), nine phosphoglucomutase (pgm, EC 5.4.2.2), six glucose-6-phosphate isomerase(GPI, EC 5.3.1.9), three mannose-6-phosphate isomerase (manA, EC 5.3.1.8), five phosphomannomutase (PMM, EC 5.4.2.8), 15 mannose-1-phosphate guanylyltransferase (GMPP, EC 2.7.7.13), 30 Sucrose synthase, (SuS, EC 2.4.1.13), 20 Sucrose phosphate synthase (SPS; EC2.4.1.14), 44 Invertase (INV, EC 3.2.1.26), 103 hexokinase (HK, EC 2.7.1.1), and 54 Fructokinase (FRK, EC 2.7.1.4) (Table S8), were identified in *B. striata*. We have generated heatmaps of the transcriptional expression of the related genes as shown in Figure 5B and Figures S5 and S6. We further analyzed the expression levels of the Root enlargement proteins (Expansin) at different growth stages of *B. striata* (Figure S7). We found that Bs.54594.4 exhibits consistently high expression throughout the growth period, especially elevated in one-year-old *B. striata*. Bs57879 shows high expression levels in three-year-old *B. striata*, while Bs.64612.1 exhibits particularly high expression in five-year-old *B. striata*. Our study delved into both relative and absolute expression patterns, leading to the identification of key BSP-related genes potentially implicated in *B. striata* polysaccharide synthesis. These genes, previously unreported, offer insights into the biosynthetic pathway of BSPs, enhancing our understanding of this process.

## 3. Discussion

*Bletilla* is a genus of orchids comprising eight species, with five native to China. The dried tubers of *B. striata*, known as ‘baiji’ in traditional Chinese medicine, are an authentic medicinal product. In ancient Chinese medical literature, detailed descriptions of Bletilla’s morphology can be traced back to the late Han Dynasty, around 200 AD, with the descriptions closely matching those of *B. striata* [24]. However, other *Bletilla* species are also used as substitutes. *Bletilla* contains a variety of chemical components, including benzol, dihydrophenanthrene, phenanthrene, and quinone derivatives, which confer pharmacological effects such as hemostasis, anti-tumor activity, and promotion of cell growth. While wild *B. striata* resources are scarce due to low natural reproduction rate and over-exploitation [4]. Due to habitat destruction and overexploitation, *Bletilla* populations have declined, making its protection an urgent priority. Our research demonstrates the physiological characteristics and gene expression changes in wild *B. striata* orchids from different years and would provide a reference for industry development and promotion of *B. striata*.

*B. striata*, a significant Chinese herbal plant in southwest China, was studied for its growth age impact on medicinal quality. It is generally believed that the medicinal efficacy of wild *B. striata* orchids is superior to cultivated ones because plants in the wild environment tend to develop more effective components based on natural conditions, while cultivated *B. striata* orchids may be influenced by human factors, leading to a decrease in medicinal efficacy [25]. Samples collected from different growth stages in Guizhou province were analyzed for total phenols, polysaccharides, alkaloids, and total proteins. Notably, phenols rose from 0.257 to 0.368 mg/g over seven years, proteins ranged from 1.77 to 1.34 mg/g, polysaccharides decreased from 65.93 to 49.63 mg/g, and alkaloids fluctuated between 0.23 and 0.34 mg/g. Prior studies have recognized the shikimic acid, phenylpropanoid, and flavonoid metabolic pathways as crucial routes for phenolic acid biosynthesis [26]. Specifically, HBA, Dactylorhin A, Millitarine, and Coelonin were identified at concentrations of 0.793 mg/g, 7.792 mg/g, 9.447 mg/g, and 0.345 mg/g, respectively, in dried suspension cells of *B. striata* [27]. As the growth years increase, *B. striata* orchid plants begin to show signs of aging, with an excessive number of old fruits on the rhizomes, which may lead to hollowing and shrinkage, affecting the quality and yield. Zhao et al. investigated the changes in active substances, metabolism profiles, and trace elements of *Ornithogalum caudatum* at different growth stages, revealing age-related variations in key components and potential markers, and offering insights for evaluating its nutritional and medicinal value [28]. Xue et al. used advanced techniques like UHPLC-Q-TOF/MS and 1H NMR to analyze *Polygala tenuifolia’*s chemical constituents over 1–3 years of growth [28]. Zhang et al. differentiated mountain-cultivated ginseng (MCG) and garden-cultivated ginseng (GCG) of different growth years using HS-SPME-GC-MS and chemometrics, identifying unique volatile compounds and markers for evaluating ginseng quality [29]. The content of effective components such as *B. striata* orchid polysaccharides and total phenols may gradually decrease, resulting in a reduction in medicinal value. The growth time of traditional Chinese medicine plants can affect the accumulation of primary and secondary metabolites, which are responsible for the medicinal properties of the herbs. Although trends are noticeable, accurately establishing the correlation between growth years and medicinal attributes remains challenging. Further research is crucial to identify molecular markers for precise evaluation and understanding of this valuable herb’s qualities.

To explore the genetic influences on *B. striata* growth and development, we identified DEGs using stringent criteria across different growth stages. A total of 11,928 DEGs were detected, with distinct patterns of upregulation and downregulation observed. Significant DEGs were found in comparisons between 1 and 3 years, 3 to 5 years, and 5 to 7 years, showcasing dynamic gene expression changes. Additionally, pathway analysis revealed key pathways influenced by age progression, such as cyanoamino acid metabolism and starch and sucrose metabolism. The enrichment of genes in pathways like plant–pathogen interaction and plant hormone signal transduction underscored the molecular mechanisms underlying growth transitions. Transcriptomics is essential for studying traditional Chinese medicinal plants, as it uncovers genetic information, biosynthetic pathways, and gene regulatory networks, thereby providing critical scientific support for their research and development. Zhou et al. identified 66 bZIP transcription factors in *B. striata* through transcriptome, and characterized their expression patterns, stress responsiveness, and interactions, providing valuable insights into their role in stress regulation [30]. Lu et al. identified and characterized 23 SWEET genes in *B. striata*, revealing their structural features, expression patterns, and responses to abiotic stresses, providing insights into the sugar transport mechanism [31]. The GO enrichment analysis of DEGs in our study highlighted age-related shifts in biological processes, with distinct pathways enriched at different growth stages. Transcription factor analysis revealed the involvement of specific TFs, like C2H2, AP2/ERF-ERF, and NAC TFs, in regulating the plant’s growth, secondary metabolite, and development across various age stages. We also identified age-related changes in metabolic pathways, symbiotic responses, and gene expression correlations in *B. striata*, while also exploring microsatellite markers for genetic diversity and breeding programs. This research on *B. striata* rhizomes elucidated key genes driving rhizome expansion and genetic regulation, shedding light on critical pathways and gene expression dynamics crucial for understanding and advancing research on the growth processes of this medicinal plant.

Polysaccharides play critical roles as architectural components on cell surfaces, forming protective capsules, cell walls, and adhesives [32,33]. In plants, polysaccharides like cellulose, xyloglucan, and pectin are synthesized in the Golgi apparatus, where they can undergo further modifications such as acetylation, sulfation, or glycosylation before being secreted [1,2,32,33,34]. The biosynthesis of these plant polysaccharides is often facilitated by the oligomerization of the polysaccharide synthases, which can assemble into larger supramolecular complexes to promote the alignment and bundling of the synthesized polymers [32,34]. This allows the formation of fibrillar structures like cellulose and chitin fibers that provide load-bearing and stabilizing functions in the cell wall [35,36]. Our study identified 298 BSP-related enzymes, shedding light on the transcriptional expression of these genes. Heatmaps of gene expression patterns further elucidated the involvement of key genes in *B. striata* polysaccharide synthesis, offering novel insights into the biosynthetic pathway and enhancing our understanding of BSP production mechanisms. However, in this study, the wild *B. striata* orchids showed a decrease in polysaccharide content with increasing years, while the total protein and total phenol content increased, indicating that overall, the *B. striata* orchids from the 5th year had the best medicinal value. Through transcriptome analysis of *B. striata* orchid rhizomes of different ages, this research has identified numerous genes involved in the expansion development of *B. striata* rhizomes and the synthesis of *B. striata* orchid polysaccharides. This not only enriches the understanding of the polysaccharide synthesis metabolism and rhizome development of *B. striata* orchids at the transcriptional level but also provides genetic resources and a theoretical basis for the study of the synthesis metabolism and development of active components in *B. striata* orchid rhizomes. It also lays the foundation for studying the rapid growth mechanism of *B. striata* orchid rhizomes and the biosynthesis of polysaccharides.

## 4. Materials and Methods

### 4.1. Plant Sample Preparation

Wild *B. striata* orchid capsules that are sturdy, mature, and unburst were collected in May 2024 from Bijie, Guizhou (27°15′355″ N, 104°9′49″ E). The weather conditions during the research period in May 2024 in Bijie, Guizhou (27°15′355″ N, 104°9′49″ E) were characterized by an overcast sky. The study site was situated at an elevation of 500 m above sea level, comprising mountain slopes, gullies, streams, and underwood areas. The agronomic practices employed involved natural growth methods, with no artificial fertilizers or cultivation disturbances within a 5 km radius, maintaining an undisturbed and uninhabited environment conducive to observing natural ecological processes. After rinsing with running water, they were air-dried and stored in a sealed bag in a refrigerator at 4 °C for future use.

### 4.2. Total Phenol, Polysaccharose, Alkaloids, and Total Proteins Detection

Colorimetric analysis was used to detect the physiological indicators. The total phenol content was determined using the FolinCiocalteu method [37]. Under alkaline conditions, phenolic substances reduce tungstate molybdic acid to produce blue compounds, which are present at 760 nm. The standard curve was prepared using gallic acid (≥95.0%; Aladdin) as a reference, with the linear regression equation obtained as y = 5.3502x + 0.017, and an R^2^ value of 0.999, where gallic acid was the x-axis and absorbance was the y-axis. The extraction method for total phenol involved: Each 5 g sample of fresh plant material was subjected to triple extraction using 75 mL of 95% ethanol at 40 °C for 10 min. The resulting extract was evaporated under reduced pressure at 40 °C to yield the dried extract for subsequent analysis. The total phenol content was determined by referencing the standard curve.

Total polysaccharide content was assessed using the phenol-sulfuric acid method, with absorbance readings taken at 490 nm using a UV-visible spectrophotometer (Lambda 750s, PerkinElmer, MA, USA) [38]. Each 5 g sample of fresh plant material underwent triple extraction. A series of gradient solutions (0.5, 0.3, 0.1, 0.08, 0.05, 0.03, and 0 mg/mL) was prepared by diluting a 5 mg/mL glucose standard solution. These solutions were measured concurrently with the samples and a standard curve was generated, correlating concentration with absorbance values. The standard curve was prepared using glucose as the x-axis and absorbance as the y-axis. The linear regression equation was obtained with an R^2^ value of 0.999.

The Coomassie Brilliant Blue G-250 method for protein content determination is based on dye-binding. Coomassie Brilliant Blue G-250 appears red in its free state but turns blue when bound to the hydrophobic regions of proteins. Each sample (5 g) of fresh plant material was extracted thrice. The maximum absorption occurred at 465 nm for the former and 595 nm for the latter. Within a certain range of protein concentrations (0–100 μg/mL), the absorption of the protein–dye complex at 595 nm was directly proportional to the protein content, making it suitable for protein quantification. To create a standard curve, a 5 mg/mL protein standard solution was diluted with distilled water to generate gradient solutions ranging from 0.5 to 0 mg/mL. These solutions were simultaneously measured with the test sample and determine their absorbance values at a wavelength of 595 nm. Then, the equation for the standard curve was established relating absorbance values to the detected concentrations.

Alkaloids react chemically with acidic dyes to form colored ion pairs. These ion pairs can be quantitatively dissolved in certain organic solvents for colorimetric determination. Depending on the acidic dye used, the acid dye colorimetric method can be categorized into the Bromocresol Green method, the Azocarmine method [39], and the Methyl Orange extraction colorimetric method. Each sample (5 g) of fresh plant material was extracted thrice. For standard curve generation, a 2 mg/mL berberine standard solution was diluted with methanol to produce gradient standard solutions ranging from 0.7 to 0.01 mg/mL. These solutions were simultaneously measured with the test sample, recording the absorbance values as A standard. Then, a standard curve was plotted based on the relationship between concentration and absorbance values.

### 4.3. RNA Library Preparation for Transcriptome Sequencing

Plant samples were extracted by the Trizol method [40]. After successful ethanol precipitation, RNA was dissolved by adding 50 μL of DEPC-treated water. Subsequently, total RNA was identified and quantified using a Qubit fluorescence quantifier and a Qsep400 high-throughput biofragment analyzer. mRNAs were enriched for polyA tails using Oligo(dT) beads, then fragmented and reverse transcribed into first-strand cDNAs. Subsequently, strand-specific second-strand cDNAs were synthesized using dUTPs. After end repair and dA-tailing, sequencing adapters were ligated, yielding a library of 250–350 bp fragments. Following PCR amplification and purification, the library’s concentration was quantified using Qubit, and fragment sizes were analyzed with Qsep400. Finally, accurate quantification was performed via Q-PCR.

Following library validation, Illumina sequenced the libraries after pooling based on concentration and target output volume, generating 150 bp paired-end reads. The sequencing process involves simultaneous synthesis and sequencing. Fluorescently labeled dNTPs, DNA polymerase, and junction primers are added to the flow cell for amplification. Each added labeled dNTP emits fluorescence during strand extension, is captured by the sequencer, and converted into sequencing peaks via software to obtain fragment sequence information. The cDNA libraries were sequenced on the Illumina sequencing platform by Metware Biotechnology Co., Ltd. (Wuhan, China).

### 4.4. Transcriptome Assembly and Gene Functional Annotation

The transcriptome assembly utilized Trinity with default settings, adjusting min_kmer_cov to 2. Gene functional annotation involved databases like NCBI Nr, Nt, KOG/COG, Pfam, Swiss-Prot, GO, and KEGG [41,42]. DESeq2 (v1.20.0) was employed for differential gene expression analysis using a negative binomial model [43], with *p*-value adjustment by the Benjamini and Hochberg method. DEGs were identified at *p* < 0.05. ClusterProfiler assessed DEG enrichment via GO terms, with a corrected *p* value > 0.05 indicating significant enrichment. KEGG provided insights into gene functions within biological systems. Transcription factor prediction utilized iTAK for plant species. DEGs were aligned to STRING for PPI network construction. SSR analysis and primer design were performed with MISA and Primer3, respectively. Weighted gene co-expression network analysis was conducted using WGCNA [44]. This comprehensive approach integrated various tools and databases to explore gene expression, functional annotation, and regulatory networks in the analyzed biological samples.

## 5. Conclusions

Our study aimed to analyze the transcriptome of wild *B. striata* tubers across multiple years to understand the molecular mechanisms regulating polysaccharide metabolism and tuber enlargement. We collected wild *B. striata* samples of different growth ages and analyzed their chemical composition, including total phenols, polysaccharides, alkaloids, and proteins. Findings indicated that the levels of these compounds fluctuated with the plant’s growth stage. To further investigate the underlying molecular mechanisms, the researchers performed RNA sequencing and transcriptome analysis, using a comprehensive bioinformatics approach to explore gene expression, functional annotation, and regulatory networks related to the development and chemical composition of *B. striata* tubers. Our study provides insights into the genetic regulation of *B. striata* development, highlighting critical pathways and gene expression dynamics essential for further research and understanding of this medicinal plant’s growth processes.

## Data Availability

Accession to cite for these SRA data: PRJNA1218429.

## Supplementary Materials

The following supporting information can be downloaded at https://www.mdpi.com/article/10.3390/plants14050689/s1. Figure S1: Phenotype change of *B. striata* under different growth years. Figure S2: (A) Box plots of the gene expression distribution in samples. (B) Spearman correlation heat map of TPM expression among samples. (C) Principal component analysis of differential samples. (D) Heatmaps of gene clusters with the most significant differences. Figure S3: The functions of the DEGs were characterized using Gene Ontology (GO) terms (A–C). Circular plot of differentially expressed genes according to the GO terms. Circular plot of the top 8 GO terms with the smallest q-values from the three major categories of Gene Ontology (Biological Process, Cellular Component, and Molecular Function). If the number of enriched GO terms in the three major categories is less than 8, all will be displayed. GO Enrichment Circular Plot: outer circle: GO terms, different colors represent different GO categories; middle circle: number of background genes and q-value; bar length indicates gene count and enrichment significance; inner circle: proportion of upregulated and downregulated genes, red for upregulated, blue for downregulated. Specific values displayed at the bottom; innermost circle: RichFactor values, each small grid represents 0.2 in the background. Pie chart of transcription factor annotation classification statistics (D–F). (A,D) 3 years vs. 1 Years; (B,E) 5 years vs. 3 years; (C,F) 7 years vs. 5 years. Figure S4: K-means cluster analysis of different gene expression levels of *B. striata* under different growth years. Figure S5: Expression of INV and FRK genes involved in Polysaccharides biosynthesis and signaling pathways. Heatmap analysis of log2-fold changes. The list of DEGs is shown in Table S6. INV: invertase; FRK: fructokinase. Figure S6: Expression of HK genes involved in polysaccharides biosynthesis and signaling pathways. Heatmap analysis of log2-fold changes. HK: hexokinase. Figure S7: Expression of Root enlargement protein (expansin) genes involved in polysaccharides biosynthesis and signaling pathways. Heatmap analysis of log2-fold changes. Table S1: Raw data of physiological phenotype testing. Table S2: Annotation status of each database. Table S3: DEGs identified among the samples. Table S4: Gene lists of each module in WGCNA. Table S5: Gene lists of blue and brown module in WGCNA. Table S6: SSR prediction result list. Table S7: SSR primer design table. Table S8: Identification and expression levels of BSPs-related genes in *B. striata*. Table S9: Expression levels of root enlargement protein encode genes.
