# Supplementary figures and images for: Transcriptome Analysis of Wild Bletilla striata Tubers Across Multiple Years Revealed the Molecular Mechanisms Regulating Polysaccharide Metabolism and Tuber Enlargement
Source: Plants (Basel). 2025 Feb 24;14(5):689. doi: 10.3390/plants14050689 (PMC11901577; doi:10.3390/plants14050689)

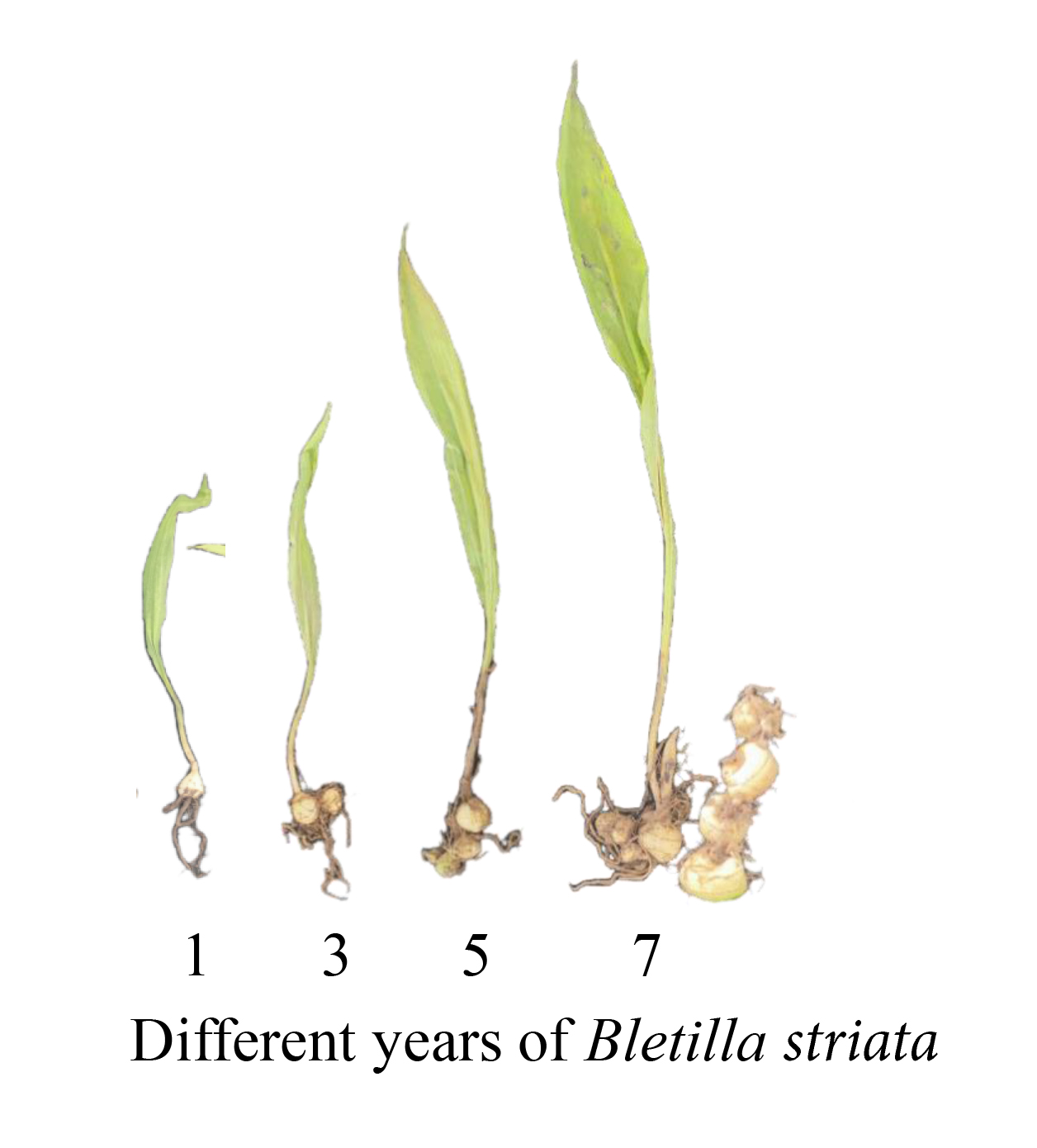

Supplement: Supplementary file 1 [file plants-14-00689-s001.zip › Figure S1.jpg]

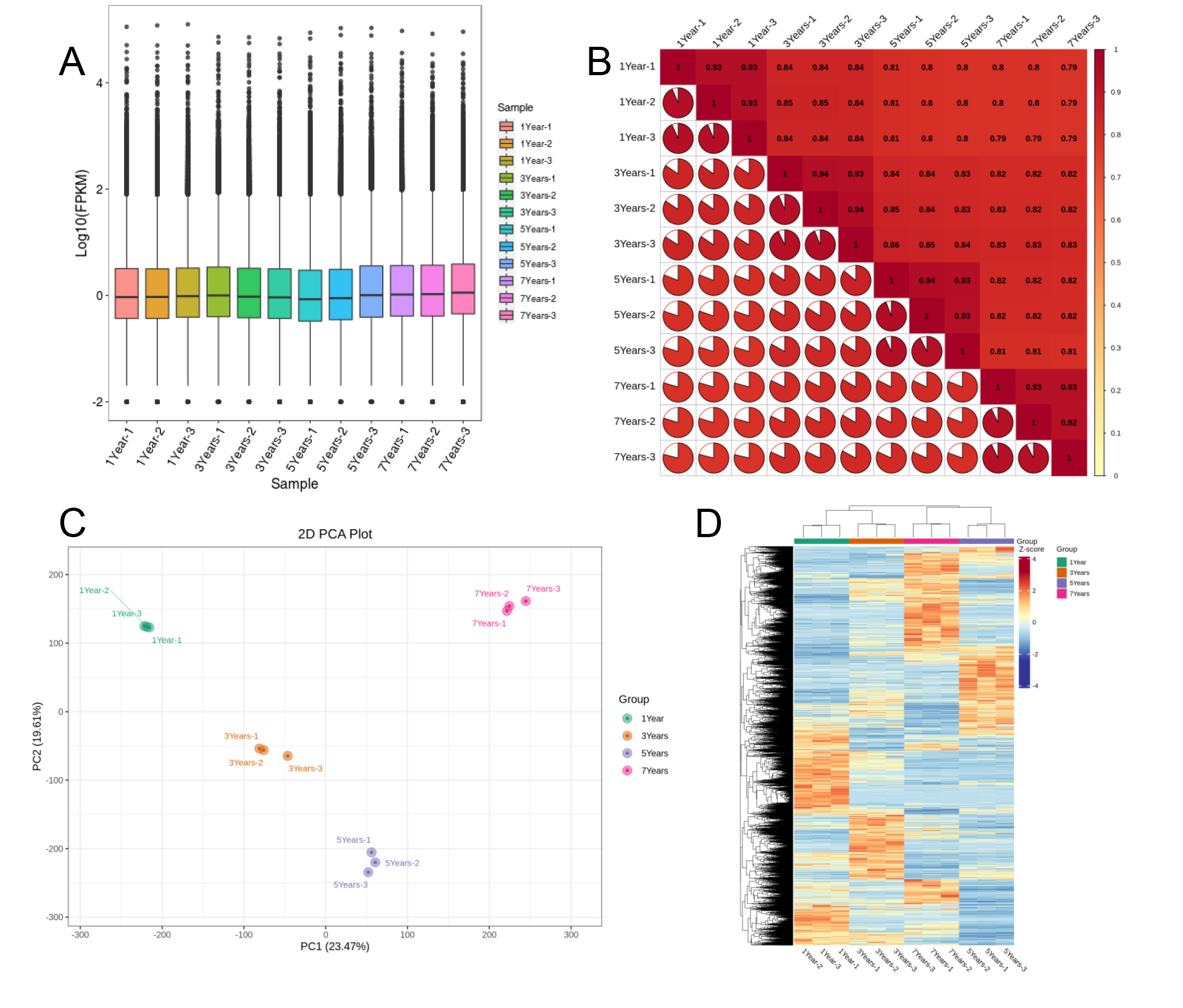

Supplement: Supplementary file 1 [file plants-14-00689-s001.zip › Figure S2.jpg]

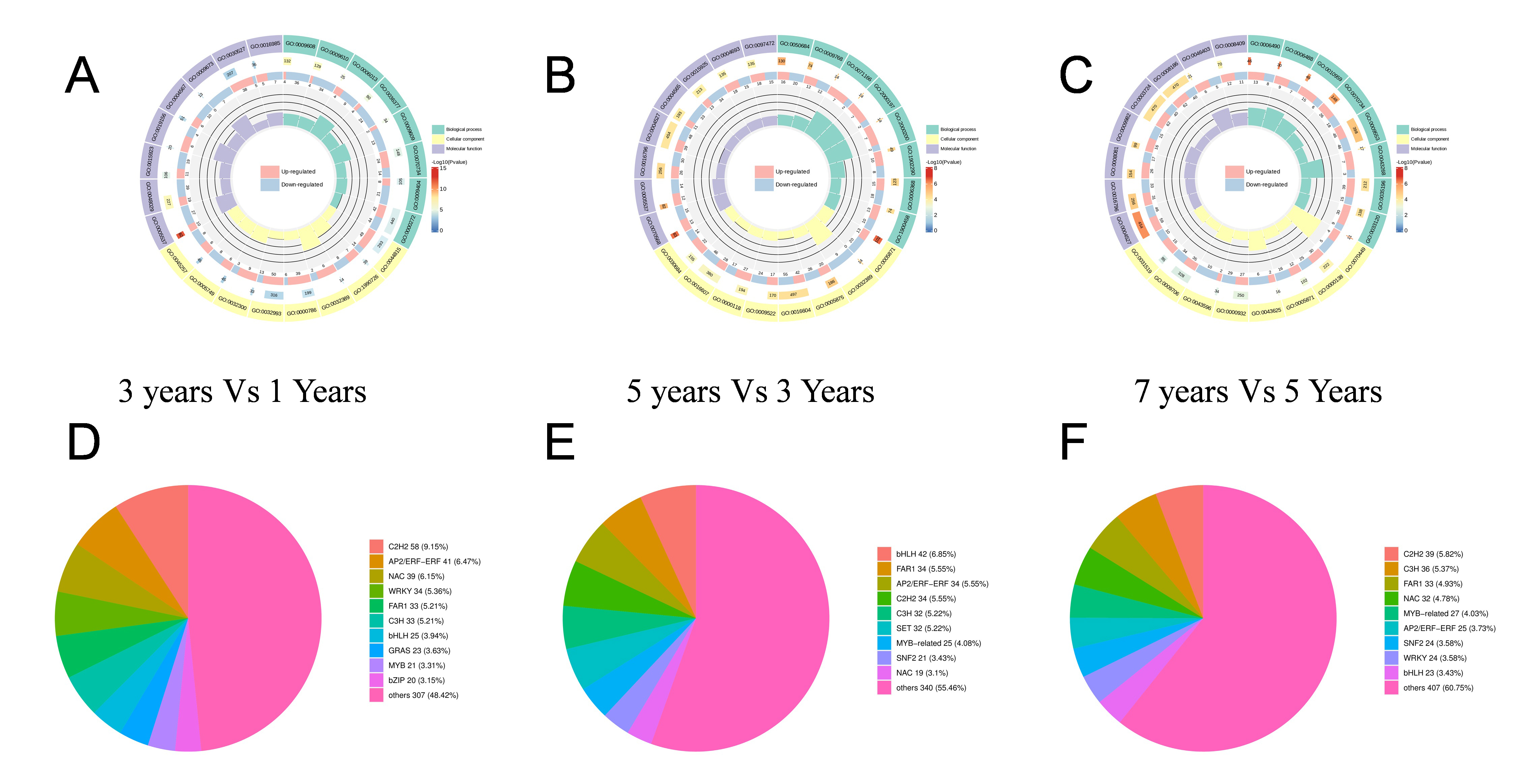

Supplement: Supplementary file 1 [file plants-14-00689-s001.zip › Figure S3.jpg]

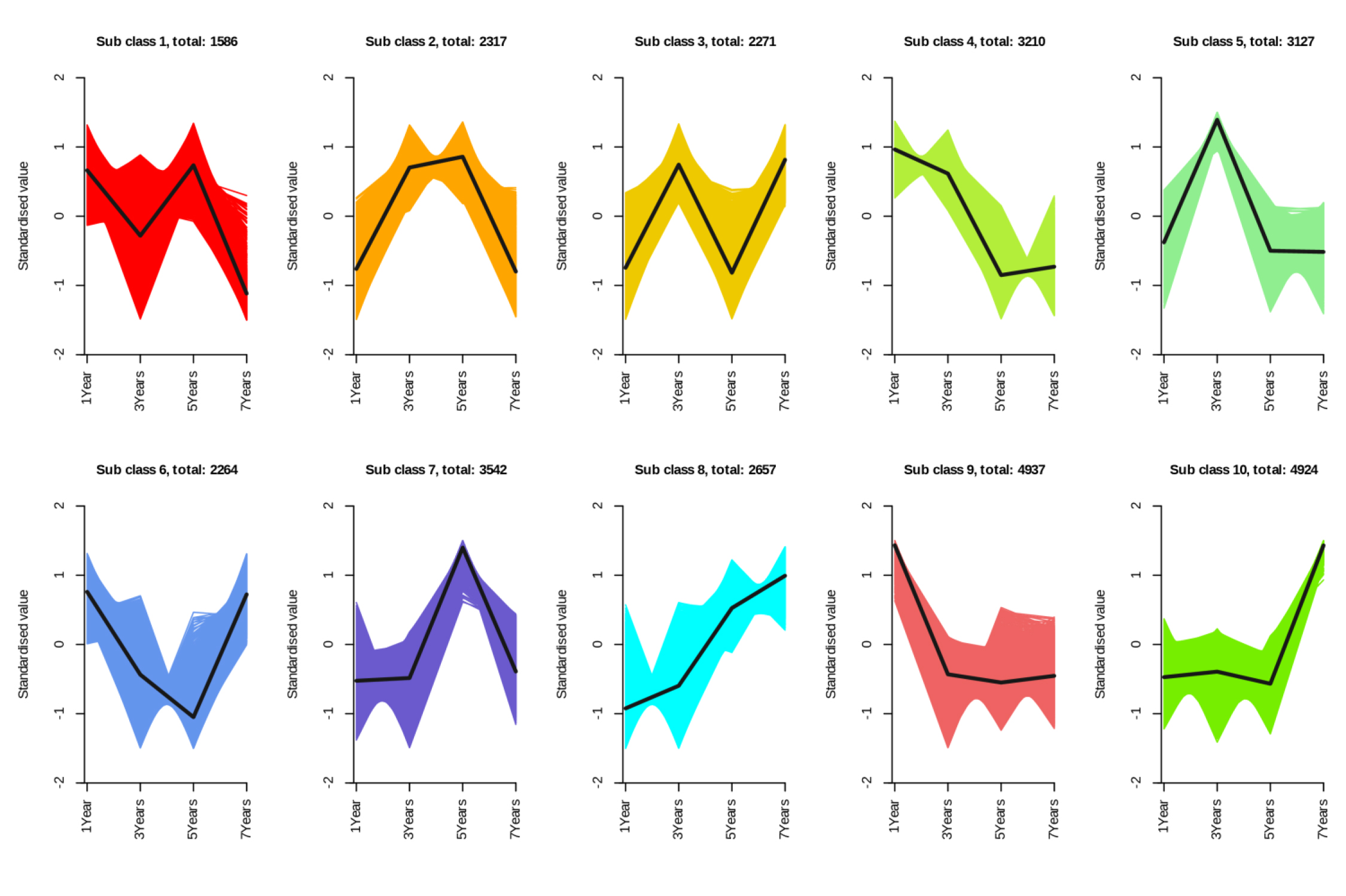

Supplement: Supplementary file 1 [file plants-14-00689-s001.zip › Figure S4.jpg]

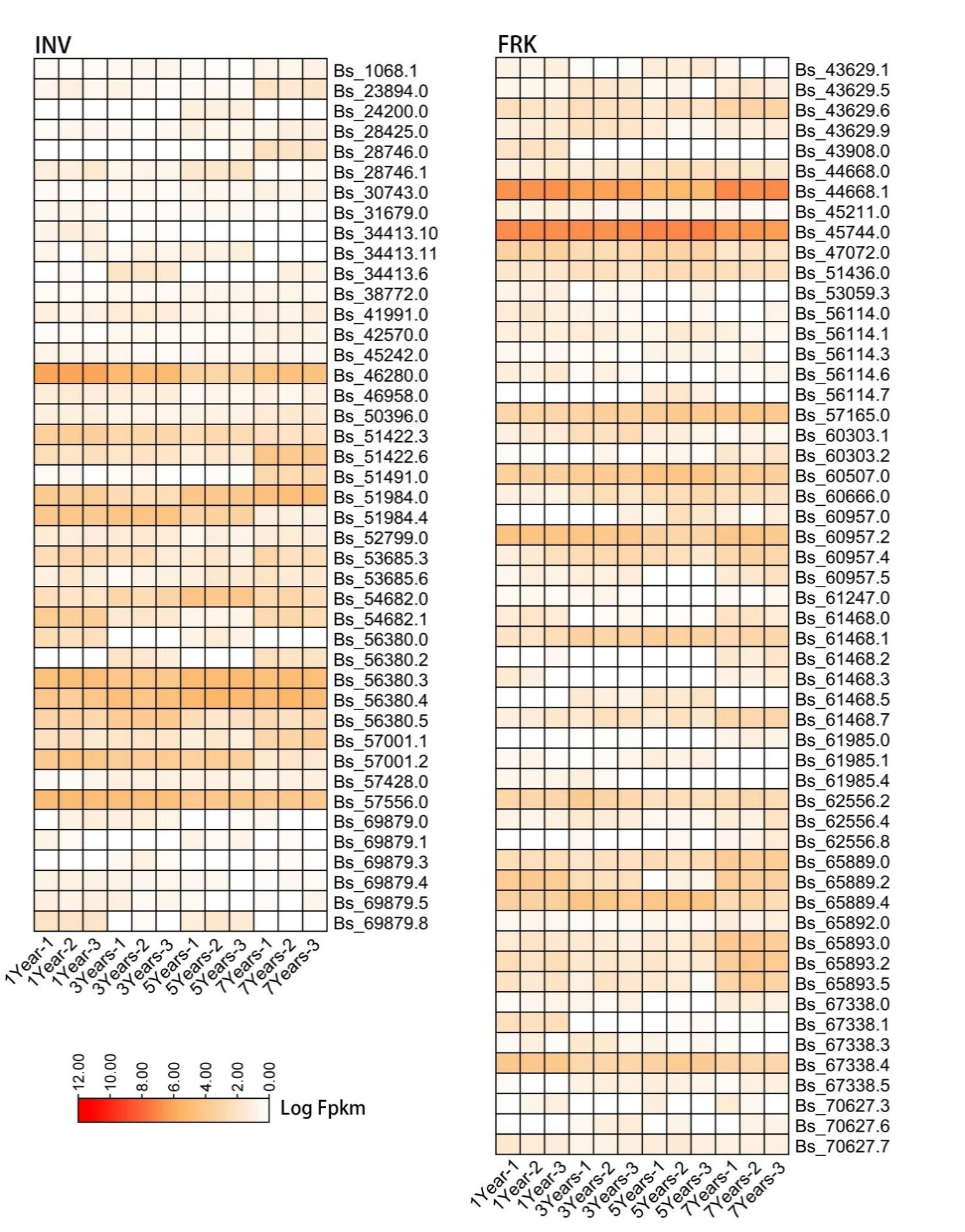

Supplement: Supplementary file 1 [file plants-14-00689-s001.zip › Figure S5.jpg]

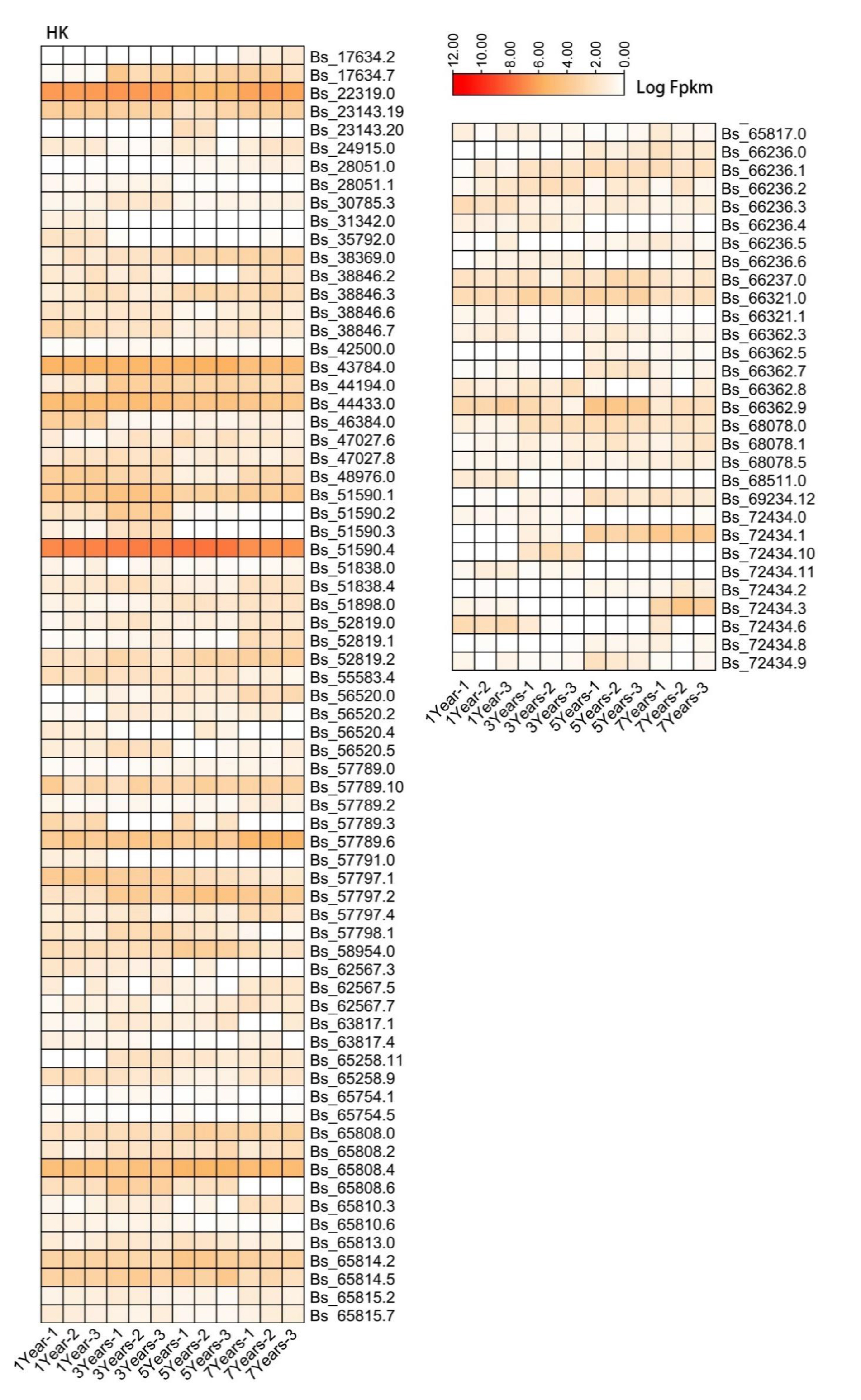

Supplement: Supplementary file 1 [file plants-14-00689-s001.zip › Figure S6.jpg]

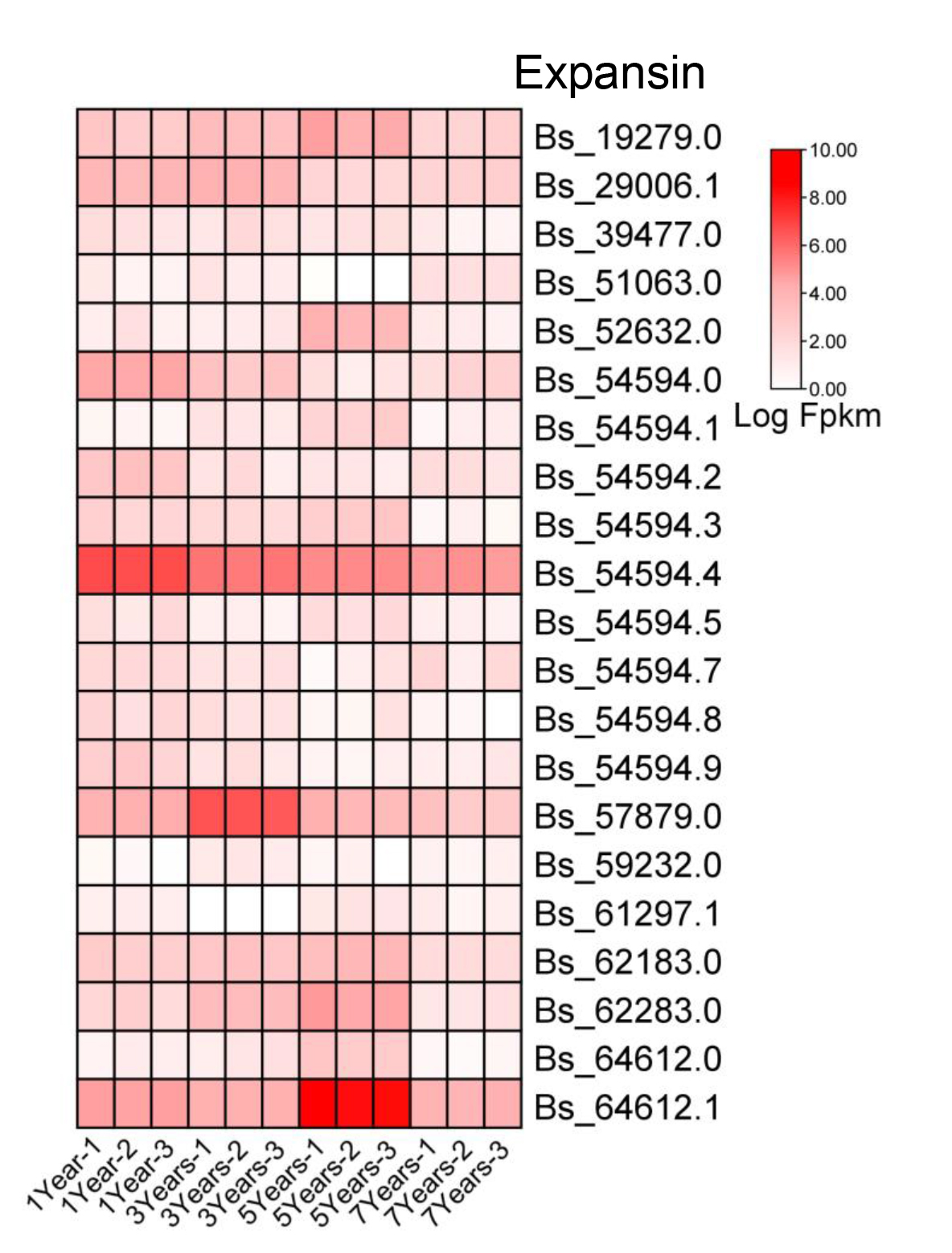

Supplement: Supplementary file 1 [file plants-14-00689-s001.zip › Figure S7.jpg]
